# Sustained release of usnic acid from graphene coatings ensures long term antibiofilm protection

**DOI:** 10.1038/s41598-021-89452-5

**Published:** 2021-05-11

**Authors:** Santosh Pandit, Shadi Rahimi, Abderahmane Derouiche, Athmane Boulaoued, Ivan Mijakovic

**Affiliations:** 1 Department of Biology and Biological Engineering, Chalmers University of Technology, Kemivägen 10, 41296 Göteborg, Sweden; 2 Department of Physics, Chalmers University of Technology, Kemivägen 10, 41296 Göteborg, Sweden; 3 Center for Biosustainability, Novo Nordisk Foundation, Technical University of Denmark, Kongens, Lyngby, Denmark

**Keywords:** Biomaterials, Nanobiotechnology

## Abstract

Protecting surfaces from bacterial colonization and biofilm development is an important challenge for the medical sector, particularly when it comes to biomedical devices and implants that spend longer periods in contact with the human body. A particularly difficult challenge is ensuring long-term protection, which is usually attempted by ensuring sustained release of antibacterial compounds loaded onto various coatings. Graphene have a considerable potential to reversibly interact water insoluble molecules, which makes them promising cargo systems for sustained release of such compounds. In this study, we developed graphene coatings that act as carriers capable of sustained release of usnic acid (UA), and hence enable long-term protection of surfaces against colonization by bacterial pathogens *Staphylococcus aureus* and *Staphylococcus epidermidis*. Our coatings exhibited several features that made them particularly effective for antibiofilm protection: (i) UA was successfully integrated with the graphene material, (ii) a steady release of UA was documented, (iii) steady UA release ensured strong inhibition of bacterial biofilm formation. Interestingly, even after the initial burst release of UA, the second phase of steady release was sufficient to block bacterial colonization. Based on these results, we propose that graphene coatings loaded with UA can serve as effective antibiofilm protection of biomedical surfaces.

## Introduction

Bacterial growth, surface colonization and subsequent biofilm formation is one of the major challenges for biomedical devices^1,2^. The biofilm formation is considered as a key factor for majority (˃ 60%) of bacterial infections such as cystic fibrosis, nonhealing wounds and medical device associated infections in humans worldwide^3,5^. The bacterial community inside biofilms represents a protected mode of survival, due to the presence of exopolymeric substances which allows bacteria to thrive in hostile environments^6–9^. Bacterial cells in biofilms are found to be 100–1000 times more resistant to antibiotics than bacterial cells in planktonic state, leading to higher risks of treatment failure by developing antibacterial resistance^10–12^. Most biomedical devices in contact with the human body are always at risk of bacterial colonization, and this leads to device-associated infections, which often lead to failure of such devices, patient suffering and financial losses^13,14^. Hence, there is significant incentive to development of antibacterial coatings to prevent or minimize bacterial attachment to biomedical surfaces.

Many nanomaterials are known to exhibit strong antibacterial effects^15,16^. For example, antibacterial activity of graphene and its derivatives has been documented extensively^17–21^. Similarly, direct antibacterial effects of metalling nanoparticles have been frequently reported. Antimicrobial activity of silver nanoparticle is widely studied and demonstrated to have strong bactericidal effect^22,23^. However, the ability of these nanomaterials and nanoparticles to protect surfaces over extended periods of time is questionable. In past decade, combining nanomaterials with antibiotics to enhance the antibacterial efficiency was intensively explored ^24–26^. Metallic nanoparticles are not particularly suitable for long-term protection, as they are not chemically inert and release metal ions that may be toxic to mammalian cells^27,28^. By contrast, graphene and its derivatives are chemically inert and possess excellent physicochemical properties for long-time protection, such as high thermal stability, biocompatibility, and high mechanical strength. Therefore, they could provide the optimal cargo system for sustained drug release to ensure long-term antibacterial protection of biomedical devices^29,30^. There are a few available studies showing the loading of antibiotics and other therapeutics to graphene derivatives, which have resulted in sustained drug release and enhancement in antimicrobial activity^31,32^. However, these tests were performed only against planktonic state of bacterial cells, which are highly sensitive to antibiotics in compared to sessile cells.

It should be mentioned that graphene coatings themselves may be engineered to provide a degree of antibacterial activity, which is based on direct mechanical interaction of bacterial cells with the exposed sharp edges of graphene^17,18^. To maximize this effect, graphene coatings/flakes need to be oriented perpendicular to the surface. Graphene coatings where flakes are predominantly arranged parallel to the surface and have no exposed edges available for interaction with bacterial cells, are typically not bactericidal^17,18,20^. Such planar or parallel-to-the-surface coatings have a high adsorption capacity, which in fact directly favours the adhesion of bacterial cells and promotes biofilm growth^33,34^.

UA is a secondary metabolites of sea lichen with strong antibacterial activity^35^. Sea lichens are surface-attached symbiotic communities composed of fungi and algae or bacteria. By producing antibacterial compounds such as UA, lichens protect themselves from colonization of surrounding bacterial communities. Considering the inhibitory activity of UA on bacterial colonization, the potential of UA to prevent surfaces of biomedical devices has already been proposed^36^. Strong antibacterial activity of UA against various human pathogens was demonstrated by previous studies^35,36^. This activity of UA is mainly based on its inhibitory potential on nucleic acid synthesis, which, via inhibiting RNA synthesis also blocks bacterial translation^35^. UA has been used as an antibacterial additive in medicinal products, toothpaste, mouth wash, wound healing patches and creams. However, poor solubility of UA limits its medical application^37^. In order to improve delivery and expand the scope of antibacterial applications of UA, this bioactive molecule has been incorporated into liposomes, nano emulsions and polymeric and metallic nanoparticles^37,38^. These strategies were effective in delivery of UA for several applications, e.g. wound treatment and targeted delivery into macrophages infected with *Mycobacterium tuberculosis*^37,38^.

In this study, we attempted to develop graphene coatings loaded with UA, which would be capable of sustained UA release and thereby providing long-term antibiofilm protection. the effectiveness of our coatings was evaluated against planktonic state and early- and late-stage biofilms of opportunistic pathogens *S. aureus* and *S. epidermidis*. These pathogens often colonise to biomedical implants, forms biofilms, infect surrounding tissues and leads towards the implant failure. We found that our coatings provide a sustained release of UA for up to one week, and we found them to be extremely effective against all forms bacterial that we tested, for periods of up to 96 h. Even after the primary burst release of UA during the initial 24 h, the coatings retained excellent antibiofilm properties. Our results indicate that this combination of graphene coatings and UA could pave the way for more effective antibacterial protection of biomedical devices, leading to diminished risks of device-associated infections.

## Materials and methods

### Bacterial strains, chemicals and materials

The graphene flakes (powder form) was purchased from Graphitene (UK). The usnic acid (UA) was purchased from Sigma-Aldrich and dissolved in DMSO. The *S. epidermidis* ATCC 35,984 and *S. aureus* CCUG10778 were used to evaluate the antibacterial and antibiofilm activity. Tryptic soy broth (TSB)/agar was used to grow bacteria.

### UA attachment to graphene

The graphene flakes were dispersed in deionized and double distilled water by ultrasonication (125 W) for 4 h at the concentration of 2 mg/mL as a stock solution. Parallelly, UA was dissolved in di-methyl sulfoxide (DMSO; 100%) at the concentration of 5 mg/mL. By mixing these stock solutions to sterile water, various concentrations of graphene—UA mixture were prepared: total concentrations of 200 µg/mL, 100 µg/mL, 50 µg/mL and 25 µg/mL. All mixtures contained equal concentrations of graphene and UA (1:1 molar ratio) in sterile water with 5% of DMSO. For example, 200 µg/mL of mixture containing final concentration of 200 µg/mL of graphene and 200 µg/mL of UA in sterile water with 5% of DMSO. After mixing, all mixtures were further sonicated for 4 h. After the sonication, graphene flakes loaded with UA were separated by centrifugation at 10,000 rpm for 15 min. The flakes were further washed twice with deionized and double distilled water. The washed flakes were mixed in original volume of sterile deionized water and further sonicated for 2 h to achieve homogeneous dispersion.

### Surface coatings and characterization

Round cover glasses (15 mm diameter) were washed with isopropyl alcohol, followed by 70% ethanol and dried by air drier. Clean and dried glass surfaces were coated with various concentrations of graphene flakes loaded with UA (25, 50, 100 and 200 µg/mL) and non-loaded graphene flakes (100 µg/mL) as control, by using a drop casting method. Specifically, 400 µL of each batch of dispersed graphene flake solution was placed on a glass surface and left overnight for complete drying at 37 °C. The dried surfaces were further cured at 60 °C for 12 h. The coated surfaces were subsequently washed with sterile deionized water to remove the flakes that were not attached to the glass surface dried at 60 °C.

The morphology of coatings was examined by scanning electron microscopy (JEOL JSM 6301F). The graphene coatings were further coated with gold (5 nm) prior to SEM imaging. Infrared spectra of the samples were recorded using an attenuated total reflection (ATR) Alpha FT-IR spectrometer from Bruker, with a diamond crystal as refractive element, in the range of 400–3000 cm^−1^ and at a resolution of 2 cm^−1^. Raman spectra were measured by a Dilor LabRAM confocal micro‐Raman spectrometer equipped with a 50 × objective and a HeNe laser (632.8 nm). Each spectrum was recorded in the range 500–3000 cm^-1^ with 10 min accumulation time and approximately 4 cm^−1^ resolution.

### Evaluation of UA release

To examine the UA release pattern from the loaded graphene flakes in the coatings, a coated cover glass was transferred to a 15 ml of falcon tube containing 1 ml of PBS buffer (pH 7.4) and kept at 37 °C. After 24 h, the buffer with released UA was collected from the tube and replaced with fresh buffer. The procedure was repeated every 24 h, for a total of 7 days. All collected samples were freeze dried and resuspended in 100 µl of milli-Q water. The amount of released UA was quantified by using HPLC (Dionex UHPLC-PDA-FLD) with the column AAA C-18 5 mm (150 mm × 4.6 mm) (AB Sciex Pte. Ltd., USA). A mixture of methanol—water—acetic acid (80:15:5 v/v) was used as the mobile phase with a flow rate of 1 mL/min. 10 µl of all the resuspended released samples and standard solutions of UA (from 1.56 to 100 µg/ml) were injected to HPLC quantified by comparison of peaks to a standard curve.

### Time kill assay

The bactericidal efficiency of UA loaded on graphene was tested against the planktonic cells of *S. aureus* and *S. epidermidis*. To prepare bacterial inoculum, single colony of *S. aureus* and *S. epidermidis* was inoculated to 5 mL of growth medium (TSB) and incubated overnight at 37 °C with continuous agitation. For this assay, 20 µl of inoculum (2–5 × 10^7^ CFU/mL bacterial cells) from overnight grown bacterial cultures were inoculated into fresh TSB medium containing sterile deionized water (control), 12.5, 25, 50, 100 and 200 µg/mL of UA loaded graphene. All samples were incubated at 37 °C with continuous agitation for 24 h. A fraction of culture (100 µL) from each sample was taken for determination of bacterial viability at time points of 0, 2, 4, 6, 8 and 24 h. The collected samples were serially diluted in 0.89% of saline. From the diluted samples, 100 µL was plated onto TSB agar plates, incubated at 37 °C for 48 h and the number of colonies was counted.

### Antibiofilm assays

The biofilm inhibitory efficiency of coatings was evaluated against *S. aureus* and *S. epidermidis*. The graphene samples loaded with UA and control glass surfaces were sterilized by UV-light exposure for 10 min. The overnight culture of respective bacteria was diluted in fresh TSB broth to obtain the final inoculum of 2–5 × 10^6^ CFU/mL and 400 µL inoculum was seeded on the pre-sterilized coated and non-coated surfaces. Samples with bacterial inoculum were incubated at 37 °C for 24 h without agitation to allow for formation of biofilms. After 24 h of growth, samples were washed twice with sterile deionized water to remove the loosely adhered or free bacterial cells and collected in 5 mL of 0.89% of sodium chloride solution. The biofilms were detached from the surface and homogenized by sonication (30 s; 10% of amplitude). The homogenized biofilm suspensions were serially diluted into 0.89% of sodium chloride solution and plated onto TSB agar plates. Agar plates were incubated at 37 °C for 48 h and the number of colonies was counted. The density of biofilms and morphology of bacterial cells in biofilms were examined by using SEM. For this, biofilms of *S. aureus* and *S. epidermidis* formed on coated and non-coated surfaces were washed twice and fixed with 3% of glutaraldehyde for 2 h. The fixed biofilms were dehydrated by using graded series of ethanol concentrations (40%, 50%, 60%, 70%, 80%, and 90%) for 15 min each, and with absolute ethanol for 20 min. The dehydrated biofilms were dried overnight at room temperature and sputter coated with gold (5 nm) prior to SEM imaging. The SEM images were acquired by using JEOL JSM 6301F (Carl Zeiss AG, Jena, Germany). To visualize the density of live and dead bacterial cells in biofilms grown on the coated and noncoated surfaces, biofilms were stained with the mixture of 6.0 μM SYTO 9 and 30 μM potassium iodide from Live/Dead BacLight Viability kit L13152, (Invitrogen, Molecular Probes, Inc. Eugene, OR, USA). Fluorescence microscopic images of the stained biofilms were acquired by using a Zeiss fluorescence microscope (Axio Imager.Z2m Carl Zeiss, Jena, Germany).

To evaluate the long-term anti-biofilm potential of coatings, biofilms were grown on the coated and non-coated surfaces for 96 h. Briefly, overnight grown bacterial suspension was inoculated on the surfaces and incubated at 37 °C for 96 h. The culture medium was replaced in the interval of 24 h (once a day, 3 times in total). After 96 h, the biofilms were collected and homogenized by means of sonication and plated on agar plates after the serial dilution to count the colonies. In addition, antibiofilm activity of coatings after the primary phase of drug release was also evaluated. In order to test this, the sterile TSB medium was placed onto the pre-sterilize coated and noncoated surfaces and incubated at 37 °C for 24 h, to allow for initial release of UA. After this incubation, the medium was removed, and surfaces were washed twice with sterile deionized water to remove any traces of the medium with released UA from the coatings. Thereafter, fresh TSB medium containing 2–5 × 10^6^ CFU/mL of respective bacterial inoculum was added onto the surfaces and incubated at 37 °C for 24 h. The number of viable bacterial cells in biofilms and visualization of live and dead bacterial cells were determined by using the methods described above.

### Statistical analysis

All data are presented as the mean ± standard deviation from at least three different biological replicates. Intergroup differences were estimated by one-way analysis of variance (ANOVA), followed by a post hoc multiple comparison (Tukey) test to compare the multiple means. Differences between values were considered statistically significant when the *P*-value was < 0.05.

## Results and discussion

### Large quantities of crystalline UA can be successfully loaded onto graphene coatings

**Figure 1:**
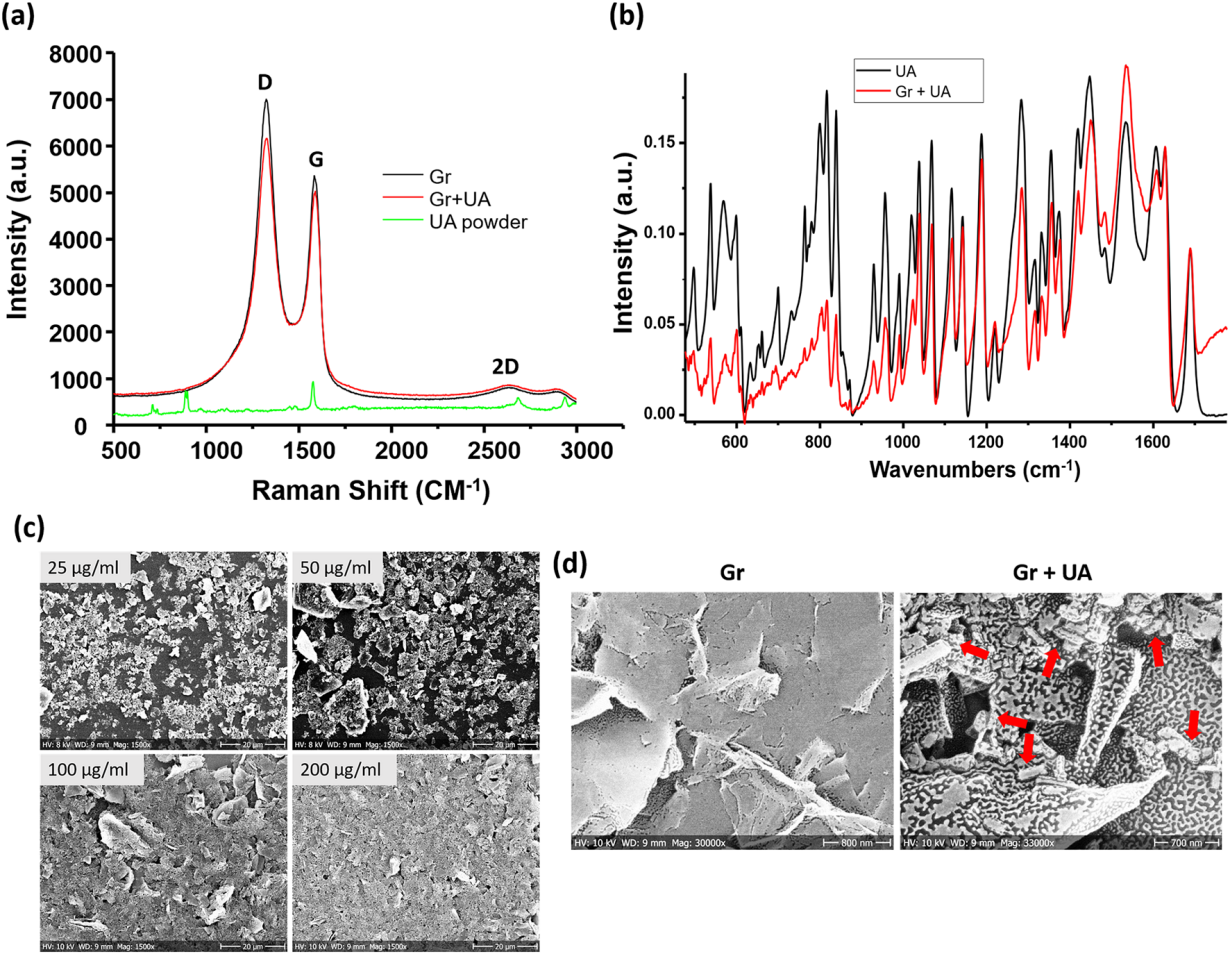
Raman spectra (**a**) and FT-IR spectra (**b**) of coatings made of graphene and graphene loaded with UA, with free UA as control. (**c**) Morphology of surface coatings with different concentrations of graphene loaded with UA. (**d**) High resolution SEM images of surfaces coated with graphene and graphene loaded with UA (100 µg/mL). The red arrow is pointing to UA in crystalline form, attached to graphene flakes.

UA was loaded onto graphene by a simple process of mixing in solution, facilitated by ultrasonication. The glass surfaces were coated with different concentrations of UA-loaded graphene. FT-IR, Raman spectroscopy and high-resolution scanning electron microscope (SEM) analysis were used to characterize the structure and morphology of the coatings and to assess the success of UA loading, as presented in Fig. 1. The usual Raman peaks of graphene were detected. The D peak (~ 1326 cm^−1^) related to defects (edges) and G peak (~ 1585 cm^−1^) from the sp^2^ C–C bond denoted the degree of graphitization of material^39^. The intensity of the D band was observed to be higher compared to the G band, indicating a considerable amount of defects and roughness of the coating surfaces. For both graphene and UA integrated samples, the minor 2D peak was identified, demonstrating the abundance of multilayer graphene in the surface (Fig. 1a)^40^.

The calculated ratio between the intensities of the D and the G bands (I_D_/I_G_) was observed to be slightly higher in the graphene loaded with UA than in pure graphene (Gr is 1.26, Gr + UA is 1.34). This ratio is known to be inversely proportional to the crystallite size of nanographite. This suggests the presence of shorter graphene sheets due to some changes in the amount of defects and hinting to sites where UA attaches.

In our FT-IR spectroscopy analysis (Fig. 1b), most peaks related to UA were clearly observed in the samples of graphene loaded with UA, with no significant peak shift. This suggests a preservation and effective integration of UA molecules with the graphene matrix. Next, the coatings of graphene and graphene loaded with UA were examined by SEM. The quantity of graphene in coatings with lower concentration of graphene loaded with UA (25 µg/mL) were insufficient to cover the surface, showing islands of graphene particles and patches of uncoated surface. With concentrations of graphene loaded with UA ≥ 50 µg/mL, the coatings were distributed homogeneously and covered the entire surface, with no significant clumping or aggregation of particles (Fig. 1c). The high-resolution SEM images of loaded and unloaded graphene coatings showed a homogenous distribution of crystalline form of UA on graphene surface (Fig. 1d), confirming successful loading.

### Release of UA loaded on graphene coatings is stable over prolonged periods

**Figure 2:**
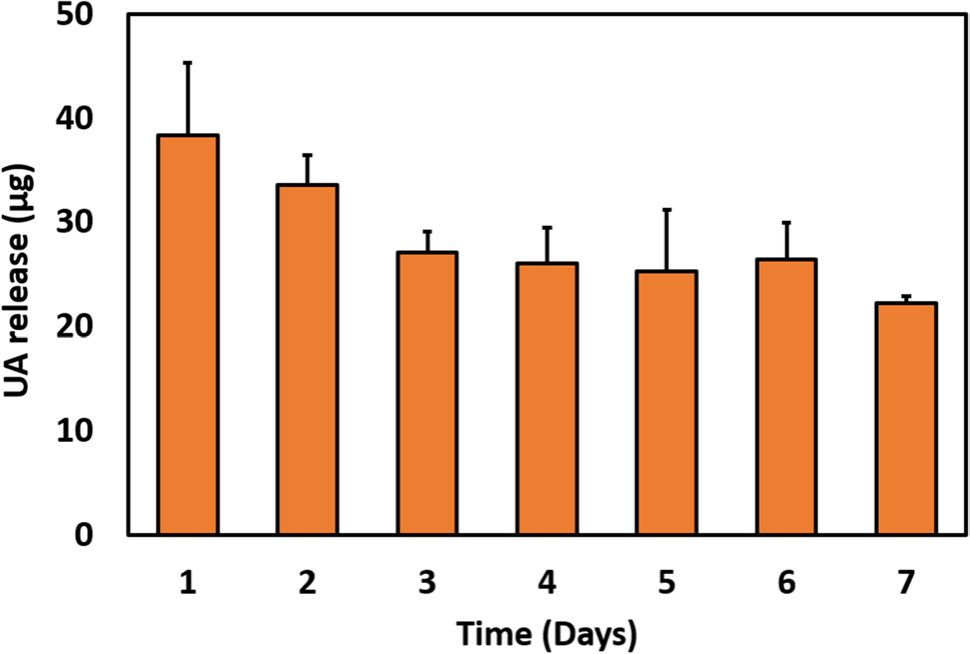
The release pattern of UA from the graphene coatings loaded with UA. The release pattern of UA was tested by using 200 µg/mL of UA loaded graphene coatings (15 mm of glass surface). The presented values are micrograms of UA detected in total volume of sample recovered from one coated surface. Data represent the mean ± standard deviation of two independent biological replicates.

Sustained release of antibacterial drugs over extended time periods is crucial for long-term antibiofilm protection of biomedical surfaces. Hence, we examined the release pattern of UA loaded onto graphene coatings for up to 7 days, using HPLC (Fig. 2). After the initial burst release, where close to 40 µg of UA were released during day 1, the release of UA remained stable at values ranging between 20 and 30 µg/day. The sustained release of UA from coatings is due to the multiple layers of graphene present on the coated surface, each of which can gradually release UA and due to the slow dissolving rate of crystalline form of UA integrated to graphene. It is previously demonstrated that integration of crystalline UA aggregates in nanomaterials slowly dissolved from coatings to medium or buffer, leading to a controlled and sustained release for longer times^41^.

### Graphene loaded with UA prevents planktonic growth of bacteria

**Figure 3:**
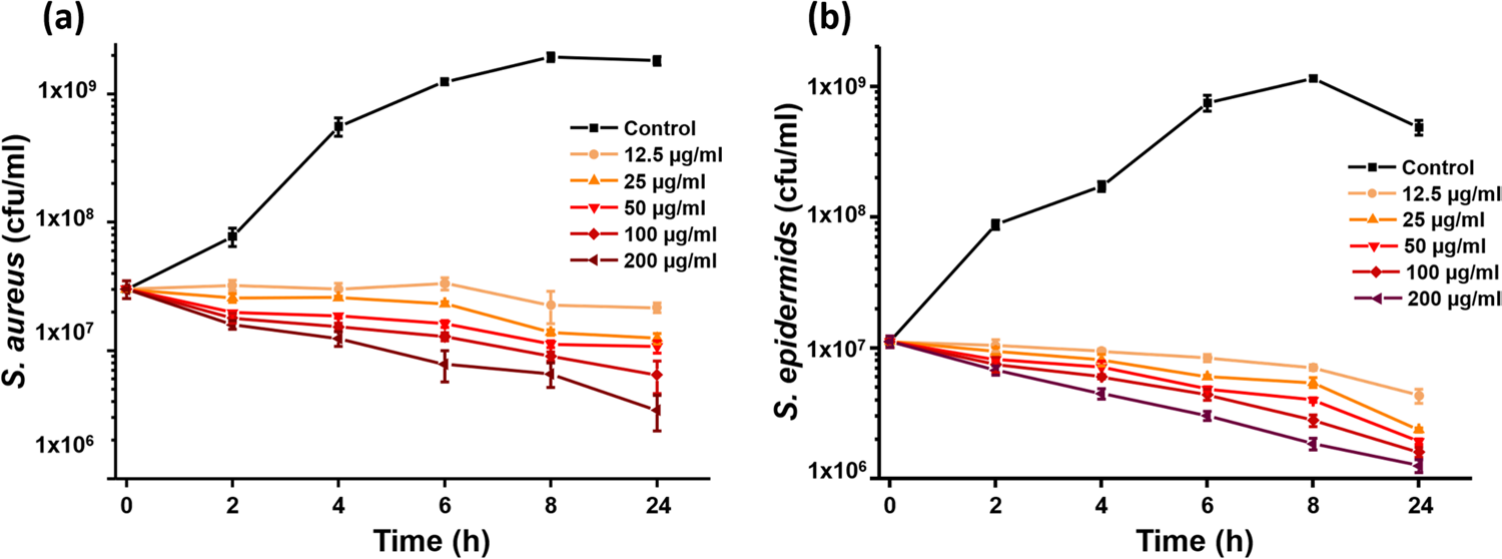
CFU counts of a) *S. aureus* and b) *S. epidermidis* exposed to different concentration of graphene-UA flakes (concentrations and colour coding are indicated in the figure inset) and grown in standard cultivating conditions. Data are presented as mean ± standard deviation from three independent biological replicates.

Antibacterial effects of our graphene loaded with UA were tested against opportunistic pathogens *S. aureus* and *S. epidermidis*. Initially, we tested the bacterial toxicity of graphene flakes loaded with UA in solution, where both the bacterial cells and the graphene-UA flakes are freely floating. Both bacterial strains were grown in the nutrient rich medium with continuous agitation (at 37 °C shaking incubator; 300 rpm), in the presence of different concentrations of graphene-UA flakes. The viability of planktonic bacteria was followed for 24 h of growth (Fig. 3).

Previous reports of UA toxicity against *S. aureus* and methicillin resistant *S. aureus* indicated maximum killing effects up to 8 h of treatment, with no further increase in bactericidal activity between 8 and 24 h^35^. In our experiments, UA loaded onto graphene flakes had more extended toxicity, with significant killing effect recorded also after 8 h (Fig. 3). The killing effect was directly proportional to the concentration of graphene-UA flakes. The lowest concentration (12.5 µg/mL) of graphene-UA flakes was bacteriostatic for the initial 2–8 h after treatment (Fig. 3a). All other concentrations were bactericidal against both *S. aureus* and *S. epidermidis* already from the first time point (2 h), and their bactericidal effects continued for the entire duration of the experiment (Fig. 3a,b). *S. epidermidis* was observed to be slightly more sensitive than *S. aureus* (Fig. 3b). Our conclusion was that graphene-UA flakes effectively prevent planktonic growth and are even capable of decimating planktonic bacteria (CFU reduction by log_10_) within a 24-h period.

### Graphene loaded with UA provides a coating that strongly mitigates biofilm formation

Adhesion of bacterial cells and subsequent biofilm formation is one of the great challenges for biomedical devices^1,2,42^. One promising strategy to reduce the adhesion of bacterial cells and prevent biofilm formation is to protect the surface of biomedical devices by various coatings^13,43^. The coating strategies reported so far include coatings containing antibiotics, containing nanoparticles with antibacterial effects, containing carbon-based materials, containing antibacterial polymers and finally, coatings made of hybrid nanomaterials loaded with antibacterial molecules^43^. So far, the major risk associated with coatings containing antibiotics/antibacterial agents is the rapid drug release, which precludes these coatings from delivering long-term antibacterial effects, and increases the risk of resistance development ^44,45^. To address this drawback, different strategies have been proposed, such as incorporation of antibiotics into a polymeric matrix, integration into nanomaterials, loading onto nanofibers followed by layer-by-layer deposition for sequential and sustained release of antibiotics/antibacterial^44,45^. Using these approaches, several classical antibiotics, such as gentamycin, vancomycin and rifampicin, have been used for implant coatings as a prophylactic measure against implant-associated infections^44,45^. An ideal coating system should be able to ensure sustained release of the drug following a two-phase profile: a first stage of burst release, followed by sustained release capable of maintain a local drug concentration above the minimum inhibitory concentration^46,47^.

**Figure 4:**
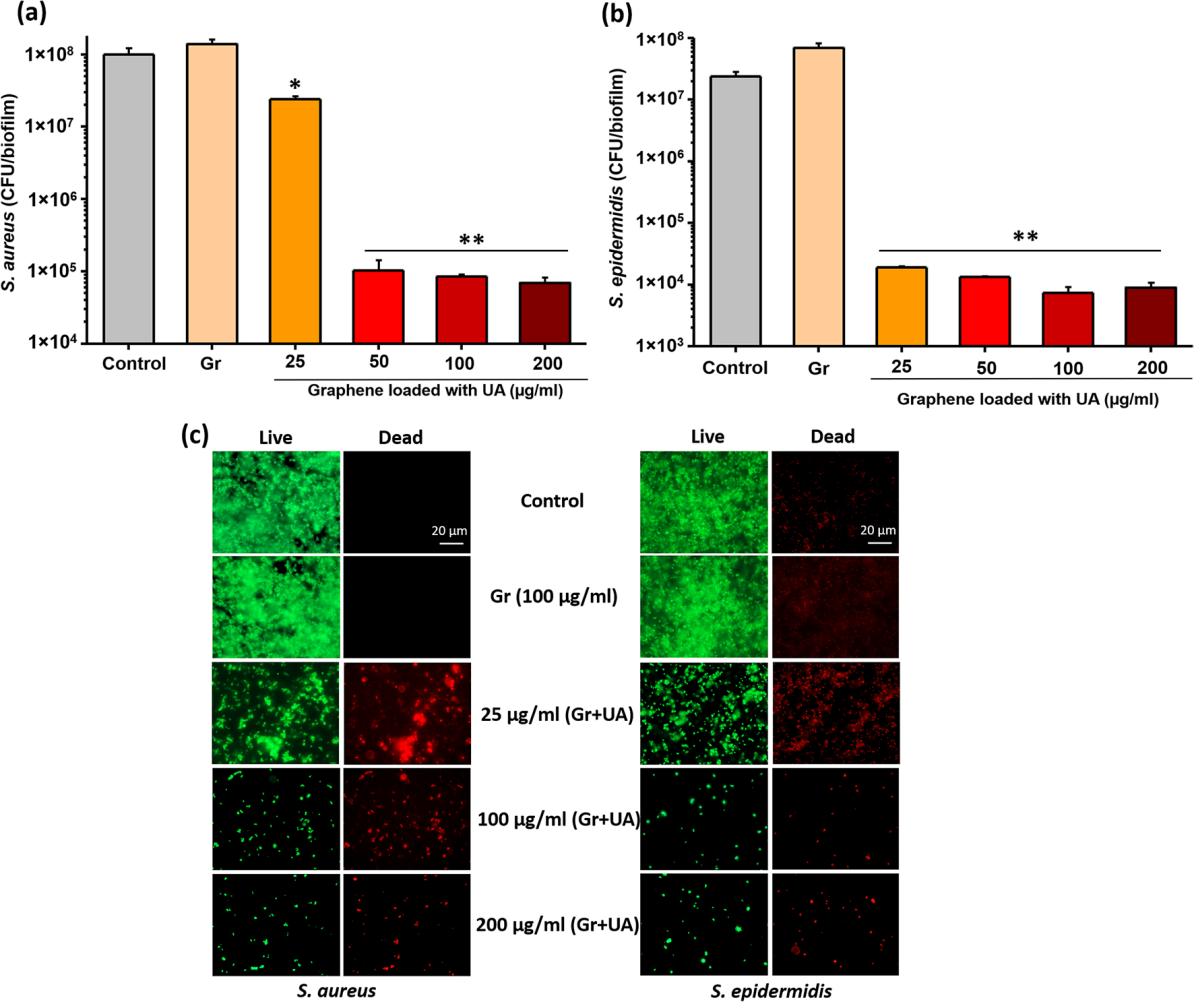
Measurement of biofilm inhibitory efficiency of graphene (Gr) and UA loaded graphene coatings against (**a**) *S. aureus* and (**b**) *S. epidermidis*. Viability of bacteria, expressed as CFU counts, was determined after 24 h of growth on coated and non-coated surfaces. Data represent mean ± standard derivation from three independent biological replicates. *P < 0.05, **P < 0.0001. (**c**) Live/dead viability staining of biofilms of *S. aureus* and *S. epidermidis* performed on the same set of surfaces as above. Representative fluorescence microscopic images from three independent biological replicates are presented. Green color denotes live bacteria and red color denotes dead bacteria.

Biofilm inhibitory efficiency of our coatings based on graphene loaded with UA was examined with the same two pathogens, *S. aureus* and *S. epidermidis*. Bacterial biofilms were grown on coated and non-coated control surfaces for 24 h. Thereafter, viability of bacteria in biofilms was evaluated by CFU counts and live/dead staining (Fig. 4). Coatings containing only graphene, not loaded with UA, exhibited no antibiofilm effects. Considering the fact that graphene flakes in the coating are mainly oriented parallel to the surface, with very few exposed edges (Fig. 1d) is not bactericidal, this result was in line with our expectations and with the available literature^17,48^. The coatings based on graphene flakes loaded with UA (Fig. 1d) inhibited formation of *S. aureus* and *S. epidermidis* biofilms by a factor of > 1000 (3 log_10_ units) at all tested concentrations (25–200 µg/mL), except for *S. aureus* at 25 µg/mL (Fig. 4a,b). Beyond 50 µg/mL, the inhibition in biofilm formation was not correlated to increasing concentration of graphene-UA flakes, indicating that a threshold concentration was achieved. A small difference in the sensitivity of *S. aureus* and *S. epidermidis* is probably due to the differences of bacterial surface, which is strain dependent. This difference is also reflected in the sensitivity of planktonic bacteria. (Fig. 3). The observed overall reduction in CFU counts (Fig. 4a,b) might be either due to the inhibitory activity on bacterial adhesion or due to the bactericidal activity of released drug from coatings. To distinguish between these two effects, we directly examined the biofilms on coated surfaces by fluorescence microscopy, after staining with live/dead viability stain (Fig. 4c). No dead cells were observed in the absence of UA, and no loss of bacterial adhesion was observed with control graphene coatings (not loaded with UA). The presence of UA in the coatings provoked appearance of dead cells (stained red), but the proportion of live/dead cells remained more or less constant with varying concentration of graphene-UA flakes. The obvious effect of increasing graphene-UA flake concentration was that of detecting fewer attached bacterial cells overall, indicating that these coatings, in addition to killing bacteria, effectively prevent early stages of bacterial colonization of the protected surface (Fig. 4c).

**Figure 5:**
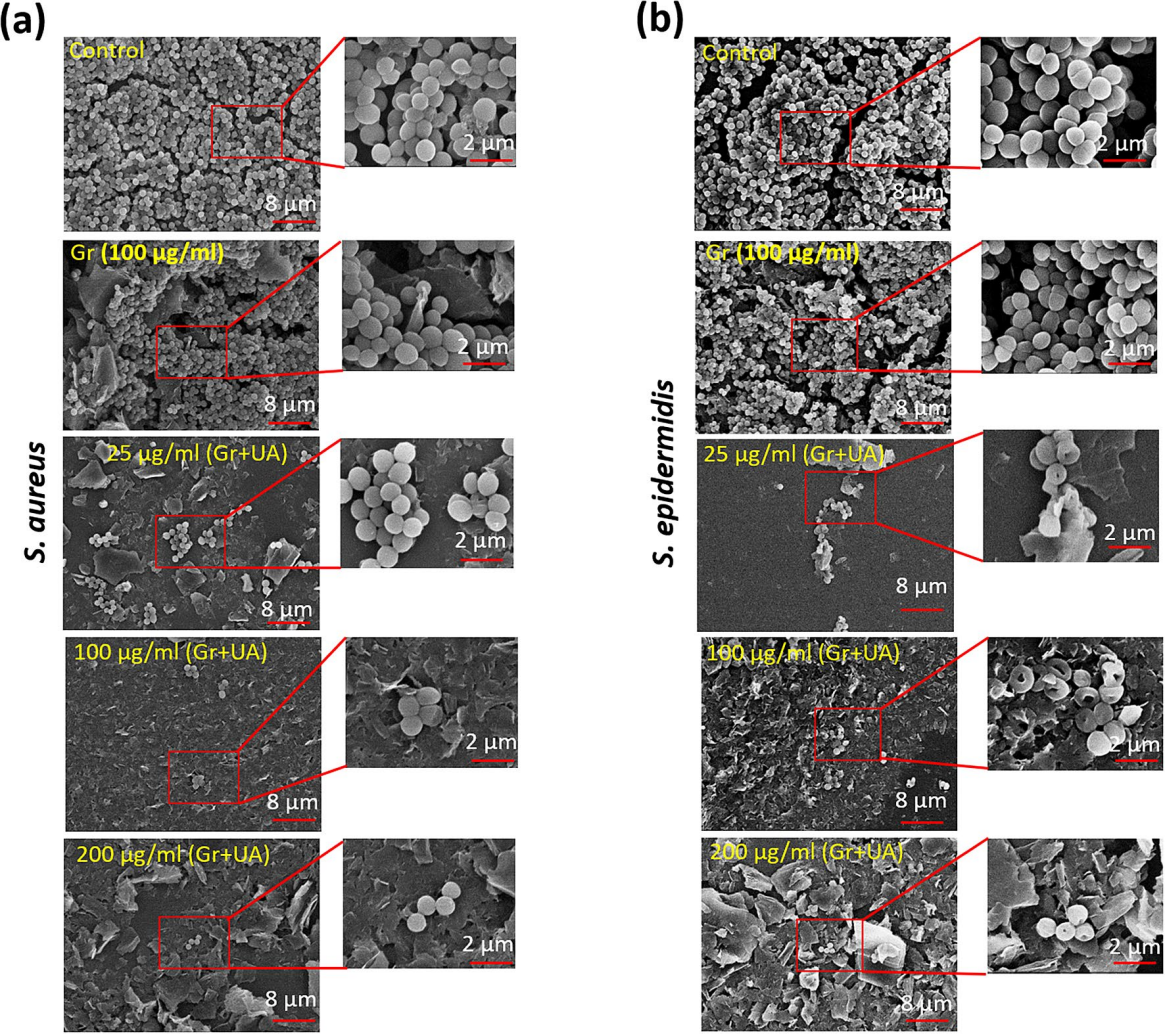
Representative scanning electron microscopic images of (**a**) *S. aureus* and (**b**) *S. epidermidis* biofilms grown on the non-coated (Control), graphene-coated (Gr) and surfaces coated with various concentrations of graphene loaded with UA. The biofilms were fixed, dehydrated and coated with gold before SEM imaging.

Further, to examine the morphological alterations by UA released from our coatings, biofilms were examined by SEM. Compared to live/dead staining, a similar trend was with the SEM analysis. Non-coated and graphene coated surfaces harboured dense biofilm structures, with some exopolysaccharide matrix (Fig. 5). Very few bacterial cells were detected on surfaces coated with graphene flakes loaded with UA. Surfaces coated with 25 µg/mL flakes harboured a slightly higher numbers of *S. aureus* microcolonies and aggregated cells (Fig. 5a), compared to *S. epidermidis* (Fig. 5b). The extent on morphological disruption of bacterial cells was more extensive in *S. epidermis*, corroborating our findings that this strain is more sensitive to UA. Overall, we conclude that three independent methods: CFU counts, live/dead staining and SEM analysis all conclusively point to a very strong inhibition of *S. aureus* and *S. epidermidis* biofilm formation on surfaces coated with graphene-UA flakes.

### Biofilm inhibition by graphene-UA coatings persists after the primary release of UA

Since our graphene loaded with UA exhibited close to ideal behaviour in terms of sustained release (Fig. 2)^46,47^, we asked whether antibiofilm effects of our coatings would also persist for longer time periods. Long-term protection is vital for surfaces of biomedical devices, because the bacterial adhesion and biofilm formation in vivo takes longer time compared to in vitro experimental setups, where large numbers of bacterial cells are inoculated and there is no competition for surface attachment by host cells^49^.

**Figure 6:**
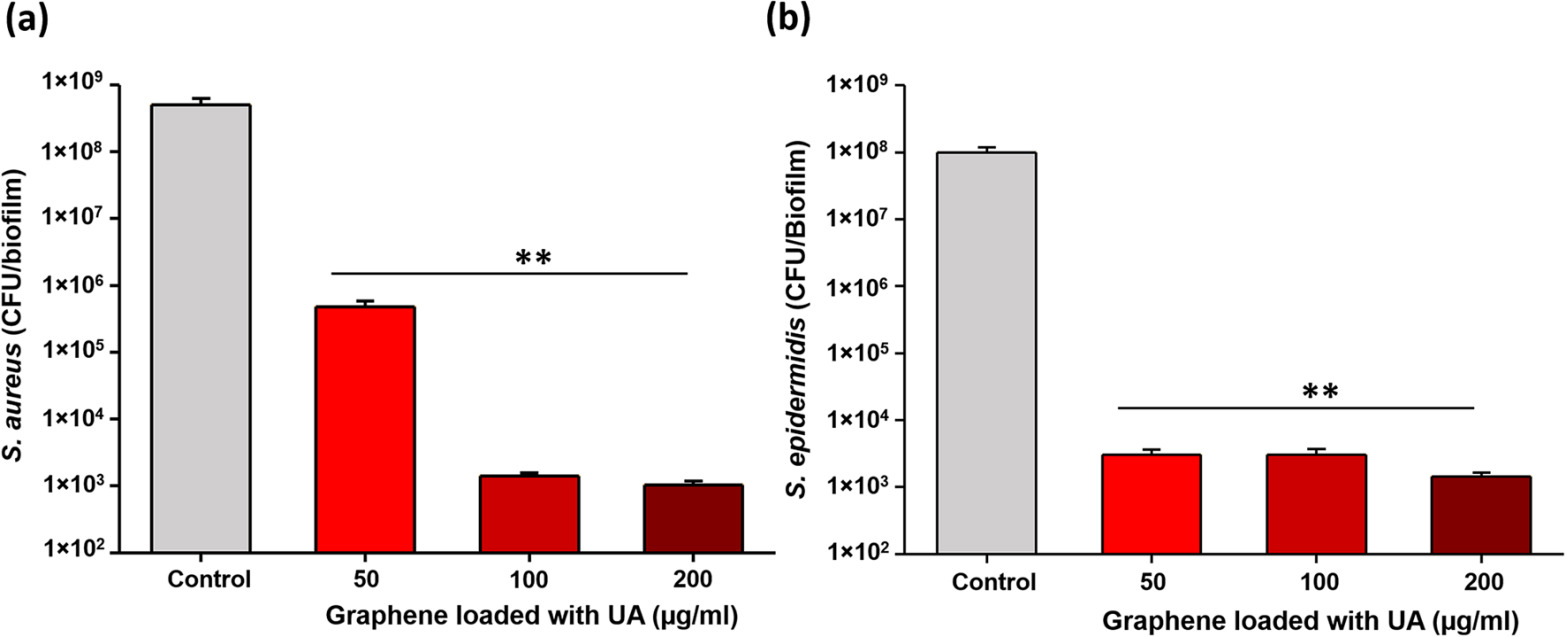
Viability of bacteria in 96 h biofilms of (**a**) *S. aureus* and (**b**) *S. epidermidis* grown on graphene-UA coatings. Data represent mean ± standard deviation from 3 independent biological replicates. **P < 0.0001.

To evaluate the long-term antibiofilm activity, viability of biofilms on various coatings was examined after 96 h, using CFU counts (Fig. 6a,b). Coatings with graphene flakes loaded with UA at concentrations ≥ 50 µg/ml dramatically reduced the viability of cells in biofilms of both bacterial species. Interestingly, the effect was much stronger than the one we measured after 24 h. Compared to a 1000-fold (3 log_10_ units) decrease at 24 h, after 96 h the decrease was closer to 100,000-fold (5 log_10_ units). This observation can be corelated to sustained release of UA (Fig. 2), which effectively prevents biofilm growth for extended time periods.

**Figure 7:**
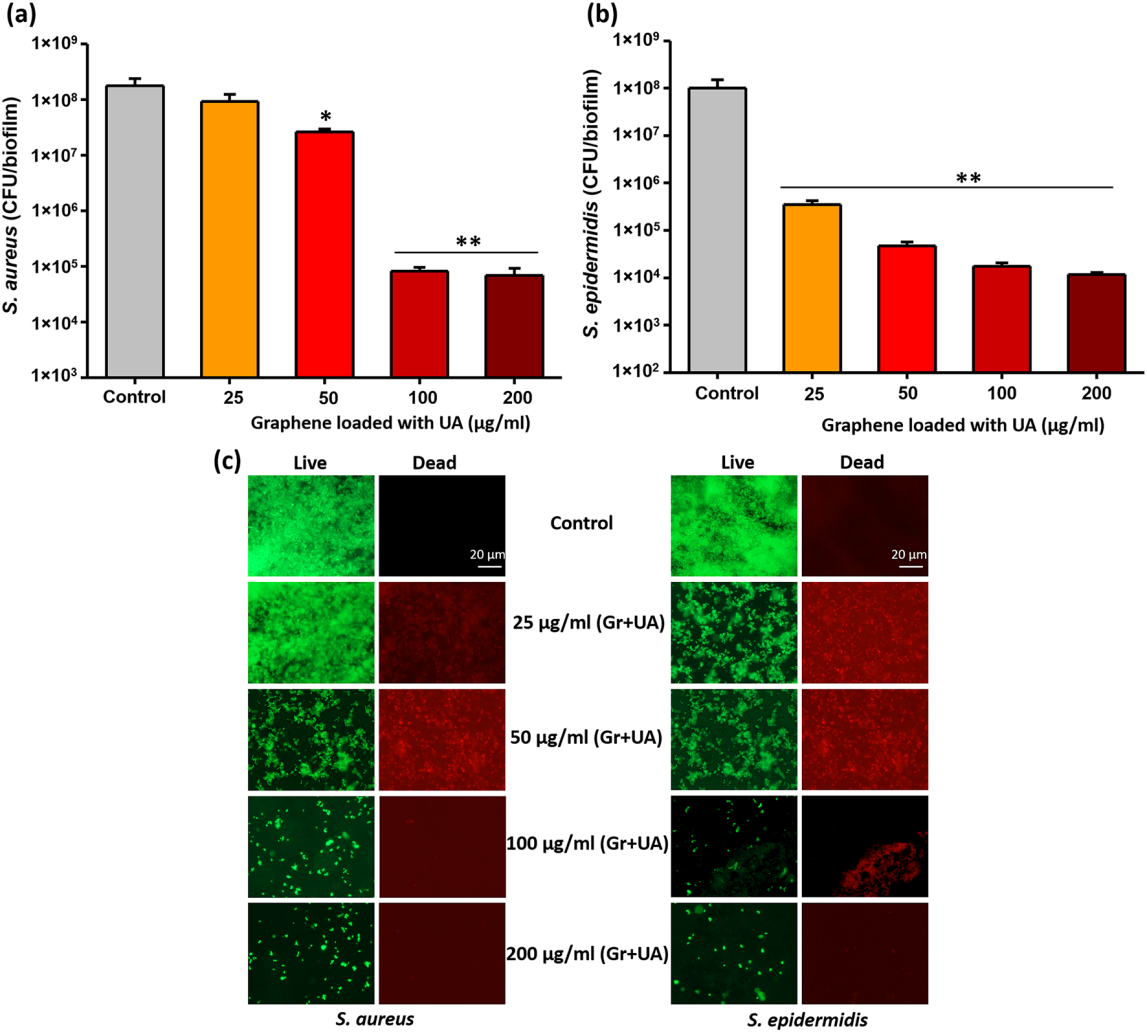
Antibiofilm activity of graphene-UA coatings. Biofilms were formed on coated surfaces after the primary release of UA. After 24 h of biofilm growth, viability of bacteria, (**a**) *S. aureus* and (**b**) *S. epidermidis*, was determined by CFU counting (*P < 0.05, **P < 0.0001) and (**c**) live/dead viability staining. Representative fluorescence microscopic images are presented from three independent biological replicates. Green color denotes live bacteria and red color denotes dead bacteria.

To further challenge our coatings, next we decided to test their antibiofilm effectiveness after completely removing the initial burst release of UA. To do this, surfaces coated with graphene flakes loaded with UA were primarily exposed to a sterile medium for 24 h. After 24 h, the medium containing primarily release UA was removed. The treated surfaces were then inoculated with a medium containing bacterial cells and incubated to allow for biofilm formation. Biofilm formation was followed by CFU counts and live/dead viability staining (Fig. 7a,b).

After the primary release, coatings with 25 and 50 µg/mL of graphene flakes loaded with UA were not able to inhibit bacterial growth to the extent that was observed when the primary release was present (Fig. 4). However, as shown in Fig. 7a,b, 100 and 200 µg/mL of coating showed full capacity for biofilm inhibition, reducing viable cell counts by > 1000-fold (> 3 log_10_ units), which was similar to inhibitory effects obtained during primary phase of UA release (Fig. 4). These results were fully confirmed by live/dead staining (Fig. 7c), where coatings with 100 and 200 µg/mL of graphene flakes loaded with UA were almost devoid of bacterial cells and harboured only small microcolonies or individual cells. Overall, our data indicate that graphene flakes loaded with UA provide coatings with excellent antibiofilm properties. They ensure sustained delivery of UA for up to 7 days, offer full antibiofilm protection up to 96 h (Fig. 6a,b) and their antibiofilm activity does not depend exclusively on the initial burst release. Rather, at flake concentrations of 100 µg/mL and above, their residual release, after removal of the burst release, is fully capable of preventing bacterial attachment (Fig. 7). The observed antibiofilm activity is solely due to the sustained release of UA from graphene coatings. The UA is widely known as a strong inhibitor of nucleic acid synthesis in bacteria by disrupting the RNA synthesis pathway^35,36^. Therefore, antibiofilm activity observed here is potentially due to bacteriostatic as well as bactericidal effect of UA by disrupting the nucleic acid synthesis pathways.

## Conclusions

In this work, we loaded graphene flakes with UA and used those to constitute surface coatings with antibiofilm properties. We demonstrated that the sustained release of UA from the coatings is the key factor in ensuring a strong and long-term antibiofilm effect of our coatings. It is difficult to correlate the findings of such in vitro studies—mimicking bacterial attachment to surfaces of biomedical devices—to real life situation in vivo. In our in vitro setup, large numbers of bacterial cells are loaded onto the surface. This is a rather advantageous situation for bacteria, compared to the in vivo conditions. In the human body, human cells compete with bacteria for attachment to the surface and the host immune system interferes with bacterial attachment^50,51^. While this makes a direct comparison between in vitro and in vivo data impossible, it should be said that a 100 000-fold reduction of biofilm growth over 96 h, as observed with our coatings in conditions optimal for bacterial attachment (Fig. 6), should be expected to translate to a very effective protection in vivo. We therefore propose that UA, loaded onto graphene coatings, could be a very effective means for preventing bacterial colonization of biomedical devices and the associated infections.
